# Biotransformation of High Concentrations of Ginsenoside Substrate into Compound K by β-glycosidase from *Sulfolobus solfataricus*

**DOI:** 10.3390/genes14040897

**Published:** 2023-04-12

**Authors:** Pan Wang, Congcong Tang, Yannan Liu, Jing Yang, Daidi Fan

**Affiliations:** 1 Shaanxi Key Laboratory of Degradable Biomedical Materials, School of Chemical Engineering, Northwest University, Xi’an 710069, China; 2 Shaanxi R&D Center of Biomaterials and Fermentation Engineering, School of Chemical Engineering, Northwest University, Xi’an 710069, China; 3 Biotech. & Biomed. Research Institute, Northwest University, Xi’an 710069, China

**Keywords:** ginsenoside, compound K, β-glycosidase, biotransformation

## Abstract

The rare ginsenoside Compound K (CK) is an attractive ingredient in traditional medicines, cosmetics, and the food industry because of its various biological activities. However, it does not exist in nature. The commonly used method for the production of CK is enzymatic conversion. In order to further improve the catalytic efficiency and increase the CK content, a thermostable β-glycosidase from *Sulfolobus solfataricus* was successfully expressed in *Pichia pastoris* and secreted into fermentation broth. The recombinant SS-bgly in the supernatant showed enzyme activity of 93.96 U/mg at 120 h when using *p*NPG as substrate. The biotransformation conditions were optimized at pH 6.0 and 80 °C, and its activity was significantly enhanced in the presence of 3 mM Li^+^. When the substrate concentration was 10 mg/mL, the recombinant SS-bgly completely converted the ginsenoside substrate to CK with a productivity of 507.06 μM/h. Moreover, the recombinant SS-bgly exhibited extraordinary tolerance against high substrate concentrations. When the ginsenoside substrate concentration was increased to 30 mg/mL, the conversion could still reach 82.5% with a productivity of 314.07 μM/h. Thus, the high temperature tolerance, resistance to a variety of metals, and strong substrate tolerance make the recombinant SS-bgly expressed in *P. pastoris* a potential candidate for the industrial production of the rare ginsenoside CK.

## 1. Introduction

Ginseng is a traditional precious herb that can be used to enhance physical vitality and treat several diseases, so it is also known as the “king of herbs”. The pharmacological activity of ginseng is mainly attributed to ginsenosides [1]. According to the literature, ginsenosides exhibit various desirable effects, such as anticancer [2], anti-obesity [3], anti-inflammatory [4], and antidiabetic [5]. Rare ginsenosides are the secondary metabolites of ginsenosides and the most important active components in ginsenosides. They have fewer glycoside groups and are more easily absorbed into the bloodstream. Compound K(CK), 20-O-β-glucopyranosyl-20(S)-protopanaxadiol, is one of the most bioactive rare ginsenosides. It has prominent anti-inflammatory [4], anti-oxidative [6], anti-diabetic [7], photoaging-protective [8], skin-protective [9], neuroprotective effects, and improving memory and cognitive dysfunction [7]. CK was also approved by the China Food and Drug Administration to be developed in clinical trials for preventing and treating rheumatoid arthritis.

CK is absent in natural ginseng plants, and is only produced by the hydrolysis of the sugar moieties in the protopanaxadiol(PPD)-type ginsenosides by the intestinal microflora of humans or rats [10]. It is currently manufactured by the deglycosylation of PPD-type ginsenosides through various means, such as heating [11], mild acid treatment [12], alkali treatment [13], and enzymatic biotransformation [14,15,16]. In addition, CK can also be synthesized by metabolically engineered yeast [17] and the nonconventional yeast *Yarrowia lipolytica* [18]. Among the above methods, enzymatic biotransformation is regarded as the principal conversion method because of its high selectivity, simple reaction steps, and mild reaction conditions [19].

Many different ginsenoside-hydrolyzing enzymes that act on different linked positions, inner and outer residues, and different types of sugar moieties in ginsenosides have been reported. To date, there have been many reports on enzymes that catalyze the conversion of major PPD-type ginsenosides into CK. Snailase, which is extracted from the digestive tract of snails, is a commercial enzyme that can convert PPD-type ginsenoside Rb1 into CK [20]. However, the high cost, low recovery efficiency, and low catalytic efficiency limit the wide production and application of snailase. Many other enzymes, such as the enzymes isolated from *Aspergillus tubingensis* [21], *Aspergillus niger* [22], and *Talaromyces purpureogenus* [23], have been reported to hydrolyze ginsenosides to CK. It is also difficult for these native enzymes to meet market demand because of their low conversion rate and yield.

The recombinant β-glycosidase from the thermophilic bacteria *Sulfolobus solfataricus*, has been reported to have broad substrate specificity and high activity for ginsenosides [24]. The thermostable biocatalyst also allows operation at high temperatures, which increases the reaction velocity and decreases contamination risk. However, the low intracellular expression and lack of post-translation modification in *Escherichia coli* may limit the large-scale application and physiochemical properties of recombinant β-glycosidase. *P. pastoris* is a eukaryotic model system for the heterologous expression of proteins. The capacities for post-translational modification and to grow high cell densities at a rapid rate make it a suitable expression system for heterologous protein production.

In this study, the eukaryotic host *P. pastoris* was selected for the efficient production of β-glycosidase from the thermophilic bacteria *S. solfataricus* (SS-bgly). Recombinase was used as a catalyst to transform the ginsenoside substrate to produce minor ginsenoside CK. The good enzymatic properties suggest that SS-bgly could be used for the industrial production of the rare ginsenoside CK.

## 2. Materials and Methods

### 2.1. Materials

*E*. *coli* TOP10 (the host strain used for vector construction) and *P. pastoris* GS115 (used for expression) were obtained from Invitrogen (Waltham, MA, USA). Restriction enzymes and ligase were supplied by Takara (Dalian, China).

Ginsenoside substrate (including 50% ginsenoside Rb1 and 15% Rd) was supplied by Zhejiang Jinai Agricultural Biotechnology Co. Ltd. (Hangzhou, China). Standards of the ginsenosides Rb1, Rd, F2, and CK were acquired from Dalian Green Biotechnology Co. Ltd. (Dalian, China). *p*-Nitrophenyl-β-D-glucopyranoside (*p*NPG) and *p-*nitrophenol (*p*NP) were acquired from Sigma (St Louis, MO, USA).

### 2.2. Construction of the Expression Strain

The codon-optimized sequence of SS-bgly (GenBank accession number WP_009992676.1) from *S. solfataricus* was synthesized by Sangon Biotech (Shanghai, China). Then, the synthetic gene was ligated to the *Kpn* I and *Not* I sites of the pPIC9K vector to obtain the recombinant plasmid pPIC9K-SS-bgly, which was stored in *E. coli* TOP10 cells. The successfully constructed plasmid was linearized with the *Sal* I restriction enzyme and electrotransformed into *P. pastoris* GS115 competent cells using an Electro Cell Manipulator (BTX, San Diego, CA, USA) at 1500 V, 200 Ω, and 50 μF. The transformants were selected on histidine-deficient MD plates at 30 °C for 2–3 days.

### 2.3. Preparation of Crude Enzyme

A successfully transformed single clone was selected, incubated in 20 mL of BMGY medium, and then shaken at 220 rpm and 30 °C until the OD_600_ reached 4–10. Cells were gathered and resuspended in 20 mL of BMMY medium, with an initial OD_600_ of 1. To induce target protein expression, 1% methanol was added every 24 h. After 120 h of methanol induction, the crude enzyme was acquired by centrifugation at 8000 rpm for 20 min. The expression of SS-bgly was evaluated by SDS-PAGE.

### 2.4. Assay of β-glycosidase Activity

The β-glycosidase activity was assayed using *p-*nitrophenyl-β-D-glucopyranoside (*p*NPG) as the substrate. The 100 μL reaction solution consisting of 80 μL of 50 mM HAc-NaAc buffer (pH 6.0), 10 μL of enzyme solution with appropriate dilution, and 10 μL of 5 mM *p*NPG was incubated for 30 min at 80 °C. The reaction was stopped by adding 100 μL of 50 mM NaOH and the absorbance was measured at 405 nm. One unit (U) of hydrolysis activity was defined as the amount of enzyme required to liberate 1 μM of *p*NP per min under standard conditions [25].

### 2.5. Enzyme Reactions and HPLC Analysis of CK

The conversion reaction was carried out in 5 mL of acetate buffer containing ginsenoside substrate and enzyme. After the reaction, methanol at 1.5 times the volume was added to the reaction system. HPLC (SSI, Philadelphia, PA USA) was used to quantitatively analyze the enzymatic reaction products at 203 nm with a C18 column. The column temperature and injected volume were 35 °C and 20 μL, respectively. The mobile phase was a gradient of acetonitrile (A) and water (B), as follows: 35% A, 0–10 min; 35% to 55% A, 10–12 min; 55% A, 12–35 min; and 55% to 100% A, 35–40 min. The flow rate was set to 1.5 mL/min.

### 2.6. Effects of pH, Temperature, and Metal Ions on CK Production

The effects of pH and temperature on the CK-producing activity were studied by varying the pH from 4.0 to 8.0 with a constant temperature of 80 °C, as well as varying the temperature from 50 to 90 °C with a constant pH of 6.0. The reactions were performed in 50 mM sodium acetate buffer containing 1 mL of fermentation supernatant and 5 mg/mL ginsenoside substrate for 4 h. The effects of 5 mM of different metal ions (CuCl_2_, FeCl_3_, MgSO_4_, CaCl_2_, KCl, and LiCl) on the CK-producing activity were tested. The addition of LiCl or MgSO_4_ improved CK-producing activity effectively_._ The effects of the different concentrations (0–10 mM) of LiCl or MgSO_4_ on the CK-producing activity were examined. All values were converted to a relative value, and the highest CK-producing activity was set as 100%.

### 2.7. Effects of Substrate and Enzyme Concentrations on CK Production

The effects of ginsenoside substrate and enzyme concentrations on CK production were studied by varying the ginsenoside substrate concentration from 10–50 mg/mL with 15 mg/mL enzyme and the enzyme concentration from 0 to 20 mg/mL with 30 mg/mL ginsenoside substrate, respectively. The reactions were performed at 80 °C in acetate buffer (50 mM, pH 6.0) in the presence of 3 mM LiCl for 48 h.

### 2.8. Analysis of Biotransformation Pathways

The biotransformation pathway that SS-bgly used to convert ginsenoside substrate to CK was analyzed using HPLC. The reactions were carried out at 80 °C in acetate buffer (50 mM and pH 6.0) containing 5 mg/mL ginsenoside substrate, 10 mg/mL SS-bgly, and 3 mM LiCl.

The kinetic parameters were determined by reacting with a series of concentrations of ginsenosides Rb1, Rd, and F2 (ranging from 0.5–30 mM, i.e., 0.5, 1, 2, 4, 8, 16, and 30 mM) at 80 °C in acetate buffer (50 mM and pH 6.0) within 10 min. The conversion rate was obtained quantitatively by HPLC. The values of the Michaelis constant (*K_m_*) and the maximum reaction rate (*V_max_*) were calculated by Lineweaver–Burk plots. The catalytic rate constant (*k_cat_*) was calculated from *K_cat_* = *V_max_*/[ET], where [ET] is the total enzyme concentration [26].

## 3. Results

### 3.1. Expression and Analysis of Recombinant SS-bgly in P. pastoris

**Figure 1:**
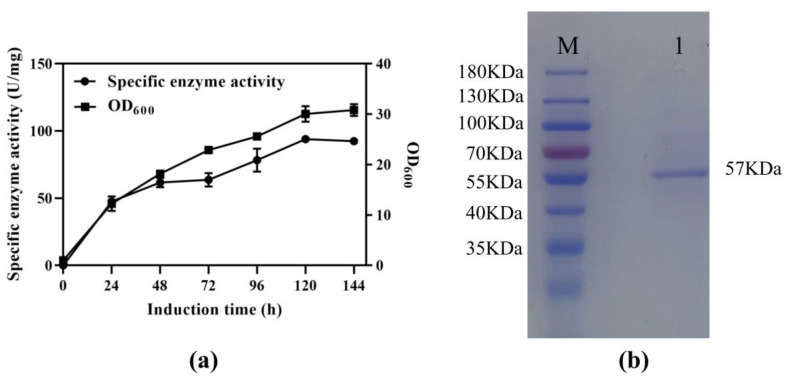
Expression of SS-bgly in *P. pastoris*. (**a**) Fermentation time curve of recombinant SS-bgly in *P. pastoris*. (**b**) SDS-PAGE analysis. Lane M: marker, Lane 1: recombinant SS-bgly.

The SS-bgly gene from *S. solfataricus*, consisting of 1470 bp and encoding 490 amino acids, was synthesized with codon optimization and ligated into the vector pPIC9K. The obtained recombinant plasmid pPIC9K-SS-bgly was verified to be correct by restriction analysis and DNA sequencing, and then transformed into *P. pastoris* GS115 for the overexpression of SS-bgly. After induction with methanol in a shake flask, the β-glycosidase activity reached the highest level of 93.96 U/mg at 120 h (Figure 1a). Cell growth was basically the same as the rate of enzyme production, which belonged to the growth-coupled type. The recombinant SS-bgly in fermentation supernatant was analyzed by SDS-PAGE and shown to have a molecular mass of approximately 57 kDa, which corresponded to the predicted value of 56,691 Da (Figure 1b) [27].

### 3.2. Effects of pH and Temperature on CK-Producing Activity

**Figure 2:**
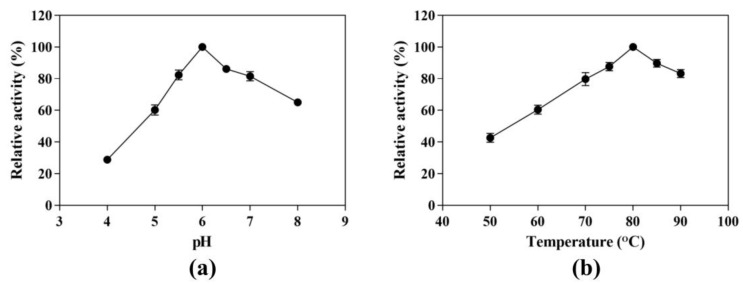
Effects of pH and temperature on the CK-producing activity. (**a**) Effect of pH on the CK-producing activity. The reactions were performed at 80 °C in acetate buffer (50 mM) containing 10 mg/mL SS-bgly and 5 mg/mL ginsenoside substrate for 4 h by varying the pH from 4.0 to 8.0. (**b**) Effect of temperature on the CK-producing activity. The reactions were performed in acetate buffer (50 mM and pH 6.0) containing 10 mg/mL SS-bgly and 5 mg/mL ginsenoside substrate for 4 h by varying the temperature from 50 to 90 °C. Experiments were performed in triplicate. The data represent the means of replicates and the error bars represent standard deviations.

We evaluated the effect of pH on CK production from ginsenoside substrate at 80 °C in a pH range of 4.0 to 8.0. The maximum yield of CK was 1.53 mg/mL observed at pH 6.0, which was set to 100% (Figure 2a). The activities at pH 5.0 or 7.0 were less than 40% and 20% of the maximum activity, respectively, indicating that the enzyme was sensitive to pH. We further detected the CK-producing activity in pH 5.5 and 6.5, and the relative activities were 82.40% and 86.13% of the maximum activity. Therefore, the optimal pH condition was still pH 6.0.

Similarly, the effect of temperature on the hydrolytic conversion of substrate to CK was investigated at 50 to 90 °C and pH 6.0 (Figure 2b). At temperatures below 80 °C, increasing the temperature enhanced the hydrolysis activity toward the ginsenoside substrate, leading to an increase in CK-producing activity. However, the CK-producing activity decreased sharply above 80 °C. In order to determine the optimum reaction temperature, we set two other temperature conditions (75 °C and 85 °C). The CK-producing activities were 87.63% and 89.73%, respectively, compared with the reaction at 80 °C. Therefore, the optimum reaction temperature was 80 °C, under which the maximum yield of CK was 1.48 mg/mL.

### 3.3. Effects of Metal Ions on CK-Producing Activity

**Figure 3:**
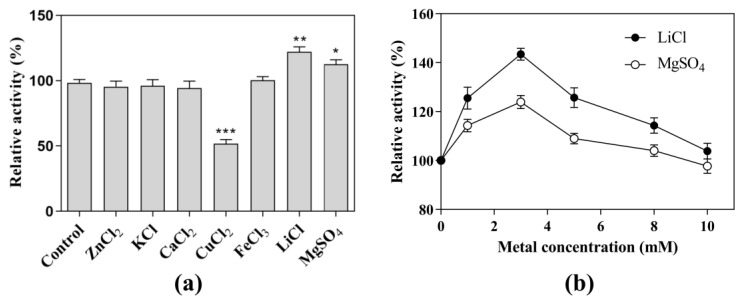
Effects of metal on the CK-producing activity. Effects of (**a**) metal ions and (**b**) LiCl and MgSO_4_ concentrations on CK-producing activity. The reactions for the effects of metal ion, LiCl, and MgSO_4_ concentrations were performed at 80 °C in acetate buffer (50 mM and pH 6.0) containing 10 mg/mL SS-bgly, 5 mg/mL ginsenoside substrate, and 5 mM of CuCl_2_, FeCl_3_, MgSO_4_, CaCl_2_, KCl, and LiCl, and 0–10 mM LiCl or MgSO_4_ for 4 h. Experiments were performed in triplicate. The data represent the means of replicates and the error bars represent standard deviations. * *p* < 0.05; ** *p* < 0.001; *** *p* < 0.0001.

Under the optimal pH and temperature conditions, each metal ion was added to a final concentration of 5.0 mM in the reaction system (Figure 3a). With the addition of ZnCl_2_, KCl, CaCl_2_, and FeCl_3_, the CK-producing activity showed almost the same levels as that without the addition of metal ions. In contrast, the addition of CuCl_2_ significantly decreased the CK-producing activity to 57.2%. The addition of LiCl and MgSO_4_ significantly increased the CK-producing activity by 25% and 15%, respectively. Further experiments showed that the activity at the optimal concentration (3 mM) of LiCl was 1.15-fold higher than that at the optimal concentration (3 mM) of MgSO_4_ (Figure 3b), indicating that LiCl was the metal salt that most effectively promoted CK-producing activity. Therefore, the next experiments were performed in the presence of 3 mM LiCl as a cofactor.

### 3.4. Effects of Substrate Concentrations on CK Production

The effect of ginsenoside substrate concentration on the production of CK was assessed using different substrate concentrations (10 mg/mL to 50 mg/mL) (Table 1). The enzyme completely converted 10 mg/mL and 20 mg/mL ginsenoside substrate to 3.79 mg/mL (12 h) and 7.58 mg/mL (30 h) CK, with productivities of 507.06 and 405.65 μM/h, respectively. As the substrate concentration increased further, the molar conversion rate decreased. When the substrate concentration was further increased to 30 mg/mL, the molar conversion rate decreased to 82.50% and reached 9.39 mg/mL CK after 48 h, with a productivity of 314.07 μM/h. However, the molar conversion rate decreased sharply as the concentration of substrate increased above 40 mg/mL. The molar conversion rates were 59.83% and 45.28% after 48 h, with productivities of 303.70 and 287.31 μM/h when the substrate concentrations were 40 mg/mL and 50 mg/mL, respectively. Under each substrate concentration, the CK production and molar conversion rates over time are shown in the Supplementary Materials, Figure S1.

### 3.5. Effects of Enzyme Concentrations on CK Production

**Figure 4:**
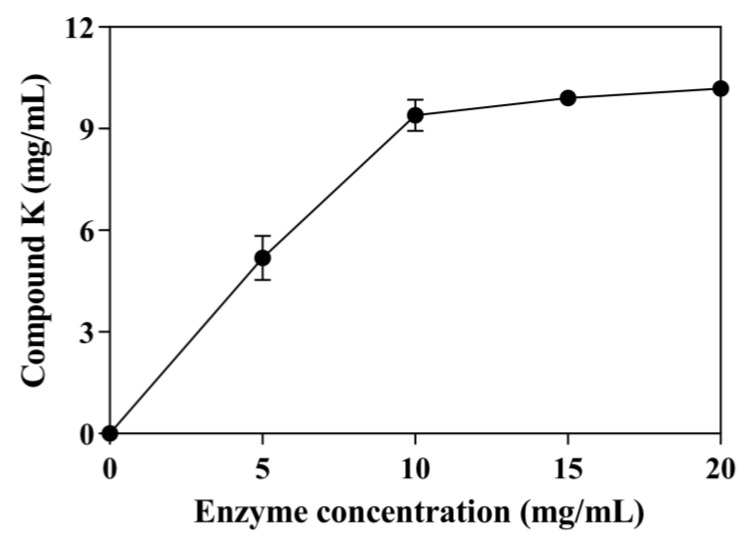
Effect of enzyme concentration on the production of CK. The reactions were performed at 80 °C in acetate buffer (50 mM and pH 6.0) containing 30 mg/mL ginsenoside substrate in the presence of 3 mM LiCl by varying the enzyme concentration from 0 to 20 mg/mL for 48 h. Experiments were performed in triplicate. The data represent the means of replicates and the error bars represent standard deviations.

The effect of enzyme concentration on CK production with 30 mg/mL ginsenoside substrate was tested using concentrations from 0.0 to 20 mg/mL (Figure 4). The conversion of ginsenoside substrate reached 45.51% using 5 mg/mL enzyme. As the enzyme concentration increased, the production of CK also increased. When the enzyme concentration reached 10 mg/mL, the conversion of ginsenoside substrate reached 82.5%, and the curve of CK production tended to be flat. Therefore, the optimal enzyme concentration was 10 mg/mL. Under these optimal conditions, SS-bgly converted 30 mg/mL ginsenoside substrate to 9.39 mg/mL CK within 48 h, with a productivity of 314.07 μM/h and a molar conversion rate of 82.50%.

### 3.6. Determination of the Biotransformation Pathway of Recombinant SS-bgly

**Figure 5:**
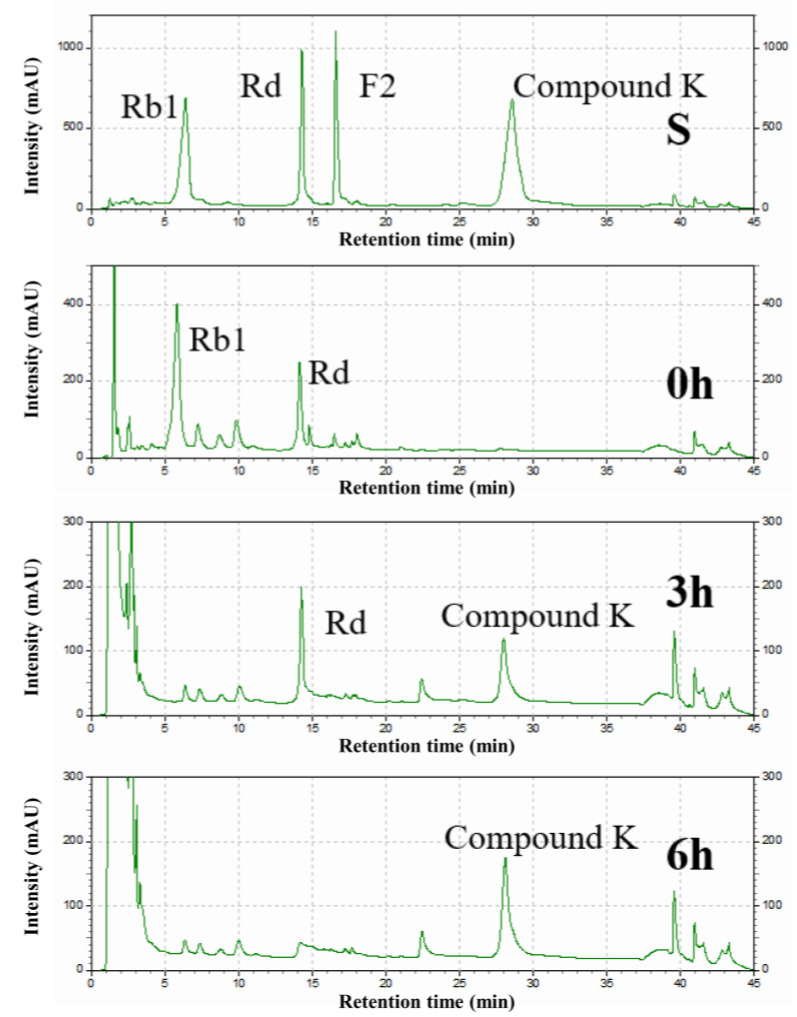
HPLC analysis of the intermediates from ginsenoside substrate into CK by the recombinant SS-bgly.

**Figure 6:**
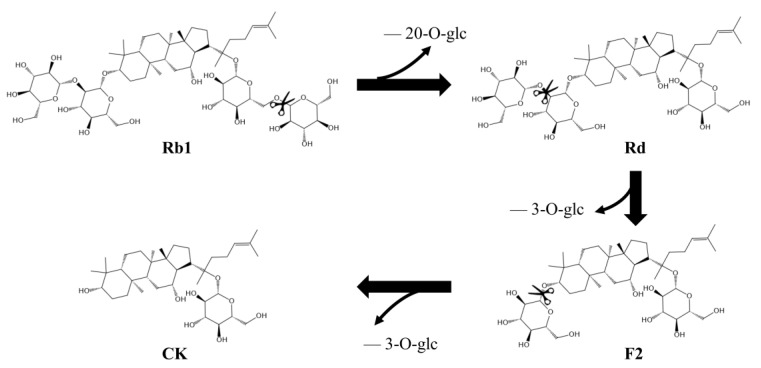
Biotransformation pathway from ginsenoside substrate into CK by the recombinant SS-bgly.

The intermediates in the conversion of ginsenoside substrate into CK by SS-bgly were confirmed using HPLC analysis (Figure 5). After 3 h, Rb1 completely disappeared, converted into 1.52 mg/mL Rd and 0.87 mg/mL CK, until 6 h, when it had been completely converted to form 1.89 mg/mL CK. The kinetic parameters for the ginsenosides Rb1, Rd, and F2 were shown in Table 2. The raw data of the kinetic parameters are shown in the Supplementary Materials, Figure S2. The *K_m_*, *k_cat_* and *k_cat_/K_m_* values followed the order of: Rd > Rb1 > F2, F2 > Rb1 > Rd, and F2 > Rb1 > Rd, respectively, which indicated that the catalytic efficiency of SS-bgly for ginsenoside F2 was much higher than those for Rb1 and Rd. We speculated that ginsenoside F2 was quickly converted into CK as soon as it appeared, so the presence of F2 was not detected by HPLC. According to the biotransformation pathway of β-glycosidase from *S. solfataricus* expressed in *E. coli* [24], we speculated that the recombinant SS-bgly hydrolyzed the ginsenoside substrate with the following pathway: Rb1 → Rd → F2 → CK (Figure 6). Ginsenoside F2 was quickly converted into CK as soon as it appeared, so the presence of F2 was not detected by HPLC. The structure of the final product was confirmed by NMR spectroscopy. The 13C-NMR and 1H-NMR results suggested that the product was CK, consistent with the results of previous reports [23].

## 4. Discussion

Recent studies have focused on the diverse pharmacological effects of the minor ginsenoside CK. However, CK is absent from the total ginsenosides. Enzymatic biotransformation is a predominant conversion modality. Several recombinant β-glucosidases have been used to convert major ginsenosides into CK, but the substrate concentrations for these enzymes are still too low for industrial applications. Among these enzymes, SS-bgly is considered to be an efficient enzyme for CK production because it can hydrolyze the different types of sugar groups. The broad substrate specificity of this enzyme sets it apart from the other reported ginsenoside-hydrolyzing enzymes. The previously reported maximal production of CK was 3.1 mg/mL by SS-bgly expressed in *E. coli* ER2566 alone, with a 75% conversion rate. While 4.2 mg/mL CK was produced by SS-bgly in combination with Cs-abf, the conversion rate increased to 100% [28]. In this study, the SS-bgly expressed in *P. pastoris* converted the ginsenoside substrate to 7.58 mg/mL CK, with a 100% conversion rate. When the substrate concentration increased to 30 mg/mL, the production of CK was 9.39 mg/mL, with an 82.50% conversion rate. Extracellular enzymes from *A. tubingensis* produced CK with the previously highest reported concentration (8.35 mg/mL) [21]. To our knowledge, the production of CK obtained in the present study by SS-bgly is the highest reported level.

To improve the conversion efficiency of ginsenosides, the reaction conditions were optimized. pH and temperature are important factors affecting ginsenoside transformation. The pH for the maximum production of CK using SS-bgly expressed in *P. pastoris* was 6.0, which was identical to that for SS-bgly expressed in *E. coli* (6.0) [28]. The temperature for the maximum production of CK was 80 °C, which was higher than those for most ginsenoside-hydrolyzing β-glucosidases. This indicates that SS-bgly is a high-temperature-resistant enzyme, which is suitable for industrial production.

Many metal ions also promote the transformation of ginsenosides. For example, Ca^2+^ contributes to ginsenoside Rd biotransformation by regulating the external calcium signals of microorganisms and by regulating β-glucosidase activity [29]. Mg^2+^ and Fe^3+^ promote the production of CK, but the specific mechanism is not yet clear [20,21]. CaCO_3_, CaCl_2_, and MnCl_2_, as metal salts, have been used to increase the activities of β-glucosidases from *Microbacterium esteraromaticum*, *Thermotoga petrophila*, and *T. purpureogenus*, respectively [23,30,31]. In our study, a series of metal ions were used to explore the conversion efficiency. LiCl stimulated the further production of CK. The promoting effect of LiCl on ginsenoside biotransformation by SS-bgly has never been reported in previous studies. We hypothesized that the addition of LiCl changed the structure of the enzyme to increase its affinity for the substrate, thus promoting the production of CK. The specific mechanism needs further analysis. Cu^2+^ could significantly inhibit the activity of SS-bgly, which was similar to that of β-glucosidase from *Aspergillus niger* CCRC 31,494 [32] and *Bacillus licheniformis* KCTC 1918 [33]. In the presence of Zn^2+^, K^+^, Ca^2+^, Fe^3+^, Li^+^, and Mg^2+^, the catalytic activity of the β-glycosidase SS-bgly was not affected or even improved. Combined with the high temperature tolerance, we believe that the recombinant β-glucosidase is an ideal candidate for complex industrial environments.

For industrialization, the substrate concentration is an important element for ginsenoside biotransformation. However, the inhibitory effect of ginsenoside substrates is a ubiquitous problem in ginsenoside biotransformation. The recombinant β- ginsenoside SS-bgly was firstly expressed in *E. coli* and converted ginsenoside Rb1 to CK with a molar conversion rate of 80% containing 1.9mg/mL Rb1 [24]. The W361F variant of SS-bgly, by using a semi-rational design, showed higher activity and catalytic efficiency, but the concentration of catalytic substrate did not increase significantly [34]. In this study, the SS-bgly expressed in the eukaryote *P. pastoris* had strong substrate tolerance. This is because, as a eukaryote, *P. pastoris* has a post-translational modification mechanism to correctly express and fold proteins. In our laboratory, Han et al. [35] screened a β-glucosidase from *Aspergillus terreus* and found that using a deep eutectic solvent (DES) combined with a continuous feeding technique could make the concentration of substrate Rb1 reach 13.3 mg/mL. In our study, the concentration of substrate Rb1 reached 15 mg/mL. To the best of our knowledge, this is the highest reported substrate concentration of ginsenoside Rb1 using recombinase. In the next experiment, we also used a cosolvent system and continuous feeding to further increase the substrate concentration.

In general, snailase is considered to be the most effective commercial enzyme for CK production, and the reported substrate concentration of ginsenoside Rb1 is as high as 70 mg/mL [20]. However, the high cost of this enzyme (2780.8 USD/kg) limits its application in industrial production. However, the crude enzyme is much cheaper and has good activity. In reference to a previous study, the cost of crude enzyme secreted by *P. pastoris* is about 27 USD/kg, which makes this method advantageous [36]. As calculated for the conversion of 1 g of ginsenoside Rb1, only 0.5 g of snailase or 1 g of SS-bgly is needed, but the latter’s cost is 25.7 times lower. Therefore, our results make the large-scale industrial production of CK possible.

## 5. Conclusions

In conclusion, in this work, we successfully expressed the recombinant SS-bgly in a *P. pastoris* expression system and explored the enzymatic reaction conditions from the ginsenoside substrate to CK. As an enzyme with strong substrate tolerance, high temperature tolerance, resistance to a variety of metals, and low cost, these properties indicate that SS-bgly may be an interesting candidate for the large-scale industrial biological production of CK.

## Figures and Tables

**Table 1:** Effect of substrate concentration on ginsenoside conversion.

Substrate Concentration (mg/mL)	CK Production (mg/mL)	Molar Conversion Rate (%)	Productivity (μM/h)	Reaction Time (h)
10	3.79 ± 0.19	100.31 ± 5.17	507.06 ± 26.11	12
20	7.58 ± 0.46	100.41 ± 6.03	405.65 ± 24.37	30
30	9.39 ± 0.19	82.50 ± 6.03	314.07 ± 6.46	48
40	9.08 ± 0.36	59.83 ± 2.38	303.70 ± 12.07	48
50	8.59 ± 0.34	45.28 ± 1.81	287.31 ± 11.42	48

Experiments were performed in triplicate. The data represent the average (±SD) of three experiments.

**Table 2:** Kinetic parameters of recombinant β-glucosidase SS-bgly for ginsenosides Rb1, Rd and F2.

Substrate	Rb1	Rd	F2
*K_m_* (mM)	3.64 ± 0.28	5.08 ± 0.32	1.86 ± 0.05
*K_cat_* (s^−1^)	20.03 ± 1.65	16.35 ± 2.26	82.42 ± 5.86
*K_cat_*/*K_m_* (s·mM)^−1^	6.25 ± 0.84	3.24 ± 0.25	42.58 ± 4.46

Experiments were performed in triplicate. The data represent the average (±SD) of three experiments.

## Data Availability

Not applicable.

## Supplementary Materials

The following supporting information can be downloaded at: https://www.mdpi.com/article/10.3390/genes14040897/s1, Figure S1. Effect of substrate concentration on the synthesis of ginsenosides. Effect of substrate concentration on the (a) molar conversion rate and (b) CK yield. The reactions were performed at 80 °C in acetate buffer (50 mM and pH 6.0) containing 15 mg/mL enzyme in the presence of 3 mM LiCl by varying the substrate concentration from 10 to 50 mg/mL. Experiments were performed in triplicate. The data represent the means of replicates and the error bars represent standard deviations. Figure S2. Lineweaver–Burk plots of recombinant β-glucosidase SS-bgly at different substrate concentrations. (a–c) Ginsenoside Rb1, (d–f) Rd, and (g–i) F2 as substrate.
