# Supplementary material for: Biotransformation of High Concentrations of Ginsenoside Substrate into Compound K by β-glycosidase from Sulfolobus solfataricus
Source: Genes (Basel). 2023 Apr 12;14(4):897. doi: 10.3390/genes14040897 (PMC10138176; doi:10.3390/genes14040897)
Supplement: Supplementary file 1 [file genes-14-00897-s001.zip › genes-2308359-supplementary.pdf]

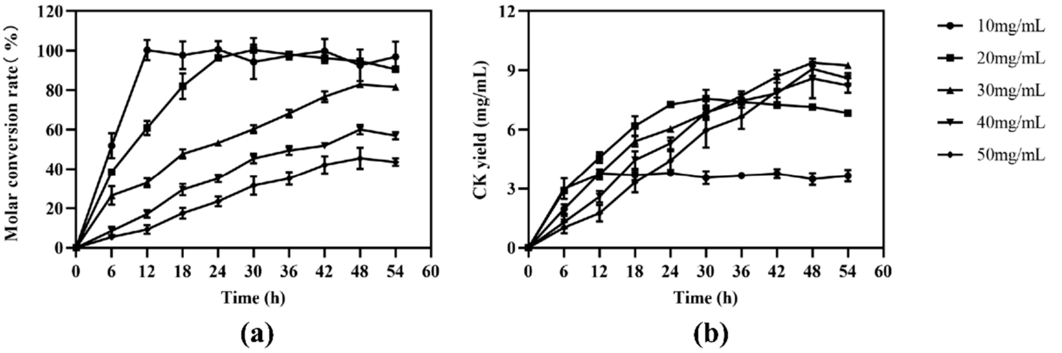

Figure S1. Effect of substrate concentration on synthesis of ginsenosides. Effect of substrate concentration on (a) molar conversion rate and (b) CK yield. The reactions were performed at 80 °C in acetate buffer (50 mM, pH 6.0) containing 15 mg/mL enzyme in the presence of 3 mM LiCl by varying the substrate concentration from 10 to 50 mg/mL. Experiments were performed in triplicate. The data represent the means of replicates and the error bars represent standard deviations.

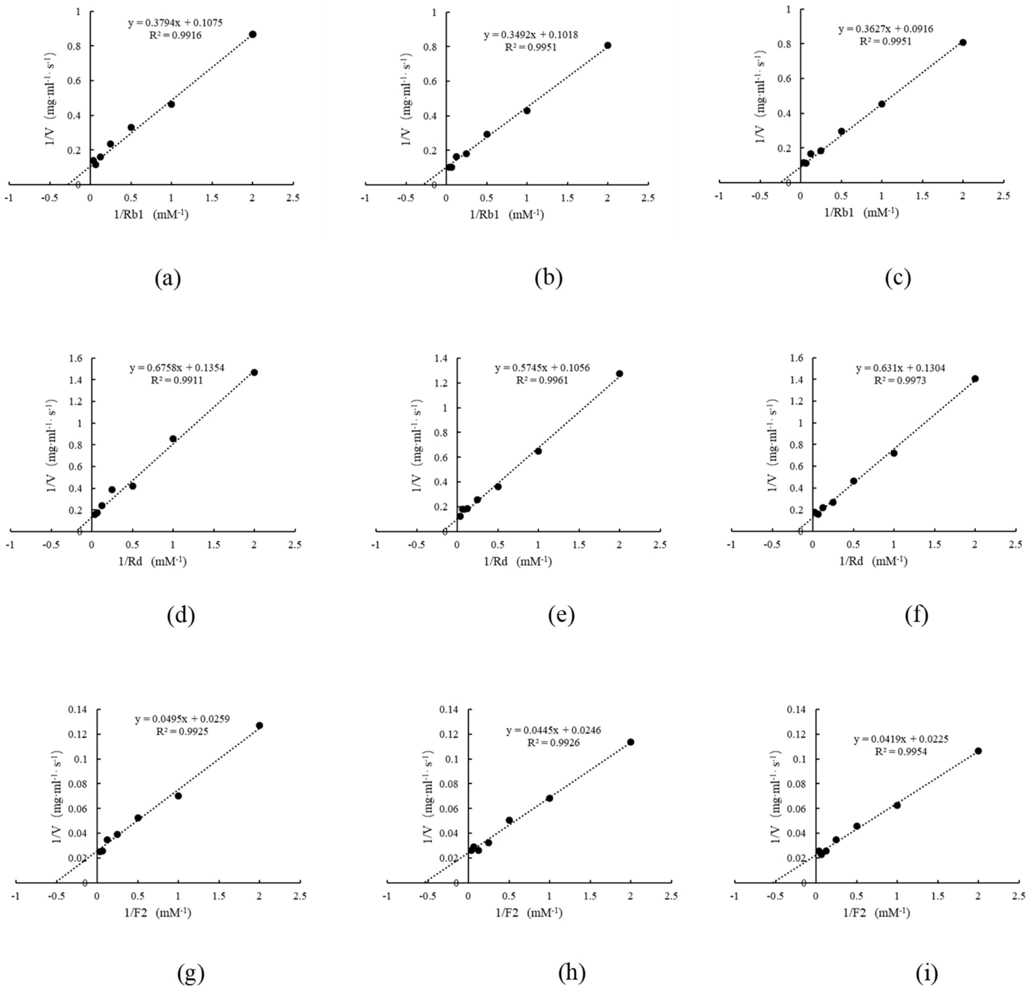

Figure S2. Lineweaver–Burk plots of recombinant β-glucosidase SS-bgly at different substrate concentrations. (a-c) ginsenoside Rb1 , (d-f) Rd, and (g-i) F2 as substrate.
